# Socioeconomic factors, attitudes and practices associated with malaria prevention in the coastal plain of Chiapas, Mexico

**DOI:** 10.1186/1475-2875-13-157

**Published:** 2014-04-23

**Authors:** Merit Mora-Ruiz, R Patricia Penilla, José G Ordóñez, Alma D López, Francisco Solis, José Luis Torres-Estrada, Américo D Rodríguez

**Affiliations:** 1 Centro Regional de Investigación en Salud Pública /Instituto Nacional de Salud Pública, Cuarta Avenida Norte y 19 Calle Poniente, C.P. 30700 Tapachula, Chiapas, México

**Keywords:** Socioeconomic factors, Attitudes, Practices, Malaria prevention, Mexico, Questionnaires, Interviews, Bed nets, Indoor residual spraying, Living conditions

## Abstract

**Background:**

Mexico is in the malaria pre-elimination phase; therefore, continuous assessment and understanding of the social and behavioural risk factors related to exposure to malaria are necessary to achieve the overall goal. The aim of this research was to investigate socio-economic backgrounds, attitudes and practices related with malaria in rural locations from the coastal plain of Chiapas.

**Methods:**

In January 2012, 542 interviews were conducted to householders from 20 villages across the coastal plain of Chiapas. Questions were about housing conditions, protection from mosquito bites and general information of householders. Chi^2^ analyses were performed to see whether there was a dependence of those reported having malaria with their house conditions and their malaria preventive practices. Results were discussed and also compared statistically against those obtained 17 years ago from the same area.

**Results:**

Most households had 2–5 people (73.6%), 91.6% of houses had 1–3 bedrooms. The physical structure of the houses consisted of walls mainly made of block or brick 72.3%, the floor made of cement 90.0%, while the roof made of zinc sheet 43.9%, and straw or palm 42.2%. A 23.1% of the interviewed completed elementary school and 16.6% was illiterate. A 9.9% of the residents reported at least one family member having had malaria. A 98.1% of families used some method to prevent mosquito bites; those using bed nets were 94.3%. Almost 72% of families bought products for mosquito protection. A total of 537 out of 542 families agreed with the indoor residual spraying (IRS) of insecticide and a frequency of application as often as every two months was preferred.

**Conclusion:**

Housing conditions and malaria preventive practices have improved in these rural areas in 17 years, which could be in favor of malaria elimination in this area. Information generated by this study could help in the decision making about whether to use insecticide as indoor residual spraying or to implement massive distribution of long-lasting impregnated bed nets, considering the number of bedrooms and the structure of houses in the region, which may lead to a more efficient vector control for the coastal plain of Chiapas.

## Background

Malaria is still a disease considered endemic in 106 countries and territories, despite control efforts implemented [1,2]. Although Mexico is in the malaria pre-elimination phase [2,3], Chiapas is considered a risk area with low, seasonal and persistent transmission [4]. During 2011, 54.1% of malaria cases in Mexico were reported in Chiapas [5], and although the cases were reduced to 32.0% during 2012, Chiapas continues being the foci with the highest malaria prevalence. Sociocultural and economic factors, such as education, housing patterns and social groups, play an important role in malaria transmission [6]. Experiences with malaria have shown that prevention is better and cheaper than cure [7].

Action plans for malaria control suggest applying indoor residual spraying (IRS) of insecticide and the use of insecticide-impregnated bed nets to prevent mosquito bites, as well as monitoring transmission mainly in areas with high levels of receptivity and vulnerability [8,9]. Questionnaires have been the tool most widely used to know factors related with malaria, such as sociocultural and demographic factors, and the information they provide helps to know and evaluate malaria control measures implemented by the control programmes, as well as to provide valuable insights necessary to guiding the implementation of malaria health promotion strategies.

In the context of the possible pre-elimination of malaria in the Mesoamerican region, and considering that vector control has been relaxed during the last decade in Mexico, it is important to determine how keen the communities are in receiving and participate with the malaria control measures if they were again implemented, as well as to know the differences in the sociocultural and demographic factors in places where the control programme used to have a continuous activity.

The aim of this study was to investigate the socioeconomic factors, attitudes and practices in the prevention of vector borne diseases like malaria, by the application of a questionnaire. Additionally, information about education levels was collected in twenty villages from the coastal plain of Chiapas, México. Responses were discussed and results compared to those given 17 years ago by villagers from the same region.

## Methods

### Study area

**Figure 1:**
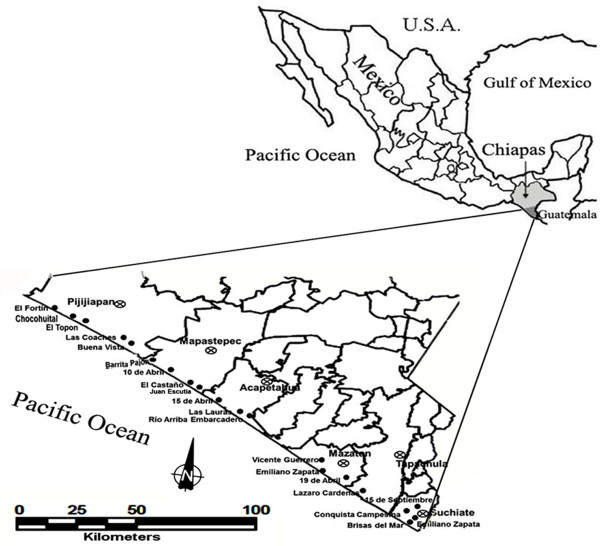
The villages selected for the study (black points), are located along the coastal plain of Chiapas, Mexico, distributed in six municipalities.

This study was conducted in the coastal plain of Chiapas, Mexico, in the same area where a similar questionnaire was applied 17 years ago [10] previously to a mosaic and rotation application trial of insecticides. Twenty villages from six municipalities were selected: El Fortín, Chocohuital, El Topón, Las Coaches and Buena Vista from municipality Pijijiapan; Barrita Pajón, 10 de Abril, El Castaño and Juan Escutia from municipality Mapastepec; 15 de Abril, Las Lauras and Río Arriba Embarcadero from municipality Acapetahua; Vicente Guerrero, Emiliano Zapata and 19 de Abril from municipality Mazatán; Lázaro Cárdenas from municipality Tapachula and; 15 de Septiembre, Conquista Campesina, Brisas del Mar and Emiliano Zapata from municipality Suchiate (Figure 1).

### Data collection

The study took place in January 2012. A questionnaire (approved by the Public Health Ethics Committee of the National Institute of Public Health, México) was administered by six trained survey personnel in a face-to-face interview with an available adult member of the family (Additional file 1 shows the questionnaire administered). Interviewers were distributed homogeneously inside the villages, so household’s information was representative. Each interviewer randomly selected the houses per block until completed the number of questionnaires assigned. According to previous studies in this area applying questionnaires [10], where at least 30% of the households were included, in this study 30 questionnaires per village was calculated to meet at least 30% of the households, except for small villages with less than 30 houses. The aim of the study was very carefully explained to the target population, and their consent individually obtained before questionnaire was administered.

The questionnaire comprised two parts: the first part was designed to collect socioeconomic status and education level of residents, and the second part about attitude and practices associated with malaria. The questionnaires were applied in every village; each interviewer administered five, except for the small villages where the number of houses was limited (10 de Abril, Mapastepec; Chocohuital, Pijijiapan; El Castaño, Mapastepec; Emiliano Zapata, Suchiate and; Vicente Guerrero, Mazatán), where less than 30 questionnaires were applied. A total of 542 questionnaires were retrieved after being administered to the residents of households, one corresponding to each family.

Data were collected in Excel 14 spreadsheets where averages and percentages were calculated. Results were described and concentrated in tables where a column with data obtained from a questionnaire taken in 1995 [10] in the same study area also was included. Chi^2^ analyses (SPSS 15.0) were performed to find out if there was association between families reporting having recent malaria and their housing conditions as their malaria preventive practices. In order to conduct the statistical analyses two indexes were created: a variable named “housing condition”, in which values from 0 to 3 were assigned to every house that met the following conditions: if cement-brick walls + cement floor + cement roof were present, the value was 3; two of any (cement walls, floor or roof) 2; one of any of the three 1; and none of them 0. The other index created was a variable named “preventive practices”, with three values assigned for the following conditions: if they reported having bed nets and purchased any product for mosquito bites prevention the value was 2; if just reported any of the two the value was 1; and if none 0. The variable “households reporting having malaria” also was created and 0 was given for those reporting no malaria and 1 for those reporting having had malaria at any time. Results were discussed and both housing conditions and preventive practices compared statistically by Chi^2^ analyses against those obtained 17 years ago.

## Results

### Socioeconomic status and education level of residents

From the total of persons interviewed, 186 (34.3%) were males and 356 (65.7%) females. Although the average of inhabitants per house was 4.34 (with a minimum of 1 and a maximum of 30 people living in a fishermen house located at Chocohuital), most households had 2–5 people (73.6%, *n =* 399), and only 2.3% (*n =* 13) had nine or more members, the percentage per number of people per house is described in Table 1. Children under five years were present in an important percentage of the families (36.2%, *n =* 196), most of them had only one child (24.7%, Table 1), but most of the families interviewed were formed by persons older than 5 years old (63.8%). A majority (91.7%, *n =* 497) of households had 1–3 bedrooms, with 40.2% of the households having one bedroom, and the average number of people per bedroom was 2.7 ± 1.6 (±SD) (Table 1).

**Table 1:** Socioeconomic and education level in the coastal plain of Chiapas between 1995 and 2012

**Questions**	**Answers**	**% 1995**	**% 2012**
Gender	Female	50.4	65.7
	Male	49.6	34.3
	4	17.9	22.5
	3	13.5	19.7
	5	20.3	18.5
Total of people living in the house	1-2	4.4	16.8
	7 and > 7	26.9	12.0
	6	17.1	10.5
	0	0	63.8
People younger than 5 years old	1	44.7	24.7
	2	41.4	9.4
	3	11.9	1.5
	>4	2.0	0.6
	1	69.5	40.2
Bedrooms	2	23.5	31.9
	3	5.0	19.6
	>4	2.0	8.3
	2	9.6	35.2
People per room in the house	1	2.4	22.5
	3	16.7	19.2
	4	23.2	10.1
	>5	45.0	12.9
	Block or brick	41.9	72.3
Walls	Palm or bamboo	20.8	15.9
	Wood	34.1	10.7
	Cement	43.3	90.0
Floor	Ground	55.8	8.8
	Zinc	13.8	43.9
Roof	Straw or Palm	51.8	42.3
	Tile	24.4	10.3
	Cement	2.6	2.6
	Others	7.2	0.9
	Housewife	49.2	65.3
Occupation	Farmer	39.2	14.4
	Fisherman	3.8	10.9
	Others	7.8	9.4
	Elementary school	13.3	23.1
	Elementary school incomplete	40.0	23.1
Schooling	Illiterate	24.1	16.6
	Middle school	3.6	14.9
	Read and write	14.3	10.3
	High school or higher	2.2	6.8
	Middle school incomplete	2.6	5.2

The physical structure of the houses consisted of walls mainly made of block or brick (72.3%, *n =* 392), palm or bamboo (15.9%, *n =* 86) and wood (10.7%, *n =* 58); the floor in most households was made of cement (90.0%, *n =* 488); while the roof was made of zinc sheet (43.9%, *n =* 229), and straw or palm (42.3%, *n =* 229) (Table 1).

Females were mostly housewives (65.3%, *n =* 354 out of the total 65.7%), while 14.4% (*n =* 78) and 10.9% (*n =* 59) out of 34.3% of the males were farmers and fishermen, respectively (Table 1). A majority of the respondents (23.1%, *n =* 125) completed elementary school (Table 1) and other 23.1% had it incomplete; 16.6% (*n =* 90) were illiterate, while just 6.8% (*n =* 37) studied high school or a higher level.

There was not a significant association between households reporting or not having recent malaria and their housing conditions according to the index which included having none, only one, any of two, or the three of the following characteristics: walls, floors and roofs made of cement or brick (Table 2).

**Table 2:** Household reporting having malaria according to housing conditions and preventive practices

**Indexes**		**Reported having malaria**		**Total**	**Chi**^**2**^
		**No**	**Yes**		**Sig.**
Housing condition	0	6.7%	0.6%	7.2%	
	1	19.5%	3.2%	22.7%	
	2	61.3%	6.3%	67.7%	
	3	2.4%	0.0%	2.4%	*P* > 0.5
Preventive practices	0	1.7%	0.2%	1.9%	
	1	25.8%	3.9%	29.7%	
	2	62.5%	5.9%	68.4%	*P* > 0.5

Housing conditions: 3 = cement-brick walls + cement floor + cement roof; 2 = any of cement walls, floor or roof; 1 = any of the three and; 0 = none of them.

Preventive practices: 2 = reported having bed nets and purchased any product for mosquito bites prevention; 1 = reported any of the two and; 0 = none.

Households reported having malaria: 1 = for those reporting having had malaria and; 0 = for those reporting no malaria.

### Attitude and practices associated with malaria

All respondents had heard of malaria and responded easily to the question about whether any family member had had malaria. Such that, 54 out of 542 (9.9%) of the residents reported at least one family member having malaria at least once in their life, and 34 out of 542 (6.3%) reported having it more than 10 year ago (Table 3). More than 98.1% of families used some method to prevent mosquito bites. Out of 542 families, 511 used bed nets (94.3%, Table 3). Ninety three percent (*n =* 477) of people who had bed nets use them all year-round and only 3.1% (*n =* 16) during the high mosquito abundance season. A total of 71.8% (*n =* 389) families bought at least one product for protection against mosquitoes. The most common product purchased was bed nets (49.4%, *n =* 268) (the other part of families only used bed nets provided by the government at not cost), while 24.2% (*n =* 131) bought mosquito coil, 23.2% (*n =* 126) bought aerosol insecticide and just 4.0% (*n =* 22) bought mosquito repellent (Table 3). A 17.5% of the total of families (*n =* 95) bought two bed nets, 12.4% (*n =* 67) bought one, 8.5% (*n =* 46) bought three, 6.5% (*n =* 35) bought four, and 3.5% (*n =* 19) bought five or more. An average of 3.7 ± 6.0 (SD) of families bought products for mosquito bites protection during the last month before the interview. Smoke from burning cardboard is used by some families to repel mosquitos indoors. Additionally, four householders referred the use of agricultural insecticides to prevent mosquito bites; two were pyrethroid-based formulations (cypermethrin and flumethrin), one organophosphate-based (parathion) and one more an organochlorine-based formulation (lindane).

**Table 3:** Vector control practices for preventing malaria in the coastal plain of Chiapas between 1995 and 2012

**Questions**	**Answers**		**1995 %**	**2012 %**
	Never		86.3	89.3
	More than 10 years ago		6.0	6.3
Households reporting at least one in the family having malaria	6-10 years ago		3.4	1.8
	2-5 years ago		2.4	1.7
	1 year ago		1.6	0.2
	Do not know		0.2	0.7
	Have bed nets		99.0	94.3
		All year	75.7	93.0
	Season usage	Rainy season	12.0	2.8
		High mosq. abundance	10.6	3.1
	Bought products in the last year		99.4	71.8
	Bought bed nets in the last year		98.2	49.4
		2	28.1	17.5
Use of products to prevent mosquito bites		1	4.9	12.4
	Number bought	3	29.1	8.5
		4	21.0	6.5
		>5	17.0	3.5
	Mosquito coil		9.6	24.2
	Aerosol		11.4	23.2
	Repellent		0.8	4.0
	>1 year ago		13.5	48.9
	Never		56.0	37.1
	1-2 months ago		2.2	4.2
The house was sprayed	3-4 months ago		8.0	4.1
	9-12 months ago		2.8	2.6
	5-6 months ago		13.9	2.4
	7-8 months ago		3.2	0.7
	Every 2 months		32.7	38.6
	Every 6 months		29.3	28.0
Frequency that would like an IRS	Every 4 months		8.4	13.3
	Each 3 months		22.7	12.0
	Yearly		5.6	7.4
	Never		--	0.7

To the question about when was their houses received the last indoor residual spraying (IRS) of insecticides, 48.9% of households responded that more than a year ago (Table 3), and although a 37.1% responded that never had their houses sprayed with insecticides a total of 537 families agreed with the IRS as a strategy to protect their houses from mosquito bites in the future, and only five respondents disagreed. Three who declined argued that IRS is dangerous for children, one more complained about the smell of some insecticides, and the last person complained about removing the furniture out before the insecticide application. From those who agreed with IRS, 38.6% (*n =* 209) preferred the application as often as every two months, 28.0% (*n =* 152) every six months, 13.3% (*n =* 72) every four months, 12.0% (*n =* 65) every three months and a few of them 7.4% (*n =* 40) yearly.

There was not a significant association between households reporting or not having recent malaria and their preventive practices which included none, any or both of: having bed nets and purchased any product for mosquito bites prevention (Table 2).

## Discussion and conclusion

Malaria is still one of the most important vector-borne diseases in Mexico and in the Mesoamerican region. Since 1955 when national malaria eradication programme was implemented in Mexico, malaria went through several stages all depending on the effort and amount of economic resources available for the intervention activities. In Mexico, as in many parts of the world, eradication was not achieved and a control programme was implemented instead. Nowadays, because of the control efforts but also due to some other external factors, malaria in Mexico is considered to be in the pre-elimination phase. Improvements in different aspects related with this programme are necessary for better results to finally achieve the eradication of malaria. The present study approaches topics related with socioeconomic factors, attitudes and practices in the prevention of malaria, how these factors have changed in time and how the changes may have contributed to maintain malaria transmission very low or with no transmission at all, even without any or very little malaria control activities in the coastal area of Chiapas.

It is well known that there are social determinants involved on health conditions [11], therefore some diseases may be associated with poverty and/or living conditions which in part may be exacerbated by cultural practices, although these cultural practices may also be so influencing, that the cycle of poverty is not broken [12,13]. In the case of malaria in México, the living conditions where extremely poor when malaria was one of the first cause of mortality and morbidity, also some demographic factors were determinants for the high transmission level. By the 50′s and 60′s, more than 75% of total population was living in rural areas [14] where educational resources and improvements in housing conditions were much lower than in cities. In those years the percentage of illiterate people exceeded the half of the population in the Chiapas state [14], a very contrasting number compared with the results of the questionnaires applied in 1995 and 2012 in the coastal plain of Chiapas, where illiteracy decreased to 24.1% and 16.6% in the families interviewed, respectively. In the same way, poor communication with cities due to lack of roads, created a problem for awareness about malaria prevention. Coupled with this, physical structure of houses was mainly made of wood, palm or bamboo, which provided an environment more conducive to the development of malaria.

At the beginning of this century, in the same study area, Rodriguez *et al.*[10] reported 43.3% of houses with cement floor and 24.89% with cement walls. That was an important change as compared to the 1950’s, however, nowadays a significantly higher percentage of houses with these characteristics (90.0% and 72.3% respectively, P < 0.01) were found in the same area. Changes in percentages of both structures have occurred after several implementation of government programmes, such as Solidaridad (2008), Programa Nacional de Desarrollo Social (2001–2006), Programa de Mejora de Vivienda by Comisión Nacional de la Vivienda (since 2001) and most recently the programmes Vivienda Rural by FONHAPO (Fideicomiso Fondo Nacional de Habitaciones Populares), programme of Promotora de Vivienda Chiapaneca part of the government programme “Chiapas Solidario 2007-2012”. These programmes gave successful support to improve the conditions of houses in rural areas during more than a decade ago. For example, houses having a single room decreased significantly from 69.5% to 40.2% from 1995 to 2012, while the percentage of houses with two and three rooms per house increased from 23.5% to 31.9% and from 5% to 19.6%, respectively (*P* < 0.01). This increment should be considered by authorities in the future, because a greater number of rooms involve higher expenditure for vector control programmes, specifically for the amount of insecticide and/or numbers of ITNs (Insecticide-treated nets) (although ITNs could represent a cheaper control option as will be further discussed). In fact, benefits of these improvements in living conditions together with the changes of socioeconomic factors in these localities could be in part responsible in the reduction of the malaria cases. For example, 2,750 cases were reported during 1994 and only 519 during 2011 in Chiapas [15], that means a reduction of five times in malaria cases in this state in 17 years.

This research showed that the use of bed nets were common in the communities studied (94.3% of the households). It is well known that the use of bed nets reduces the degree of human-vector contact and malaria transmission can be significantly lowered by the use of insecticide impregnated bed nets [8,16-18], as well as could decrease malaria to pre-elimination levels by reducing mosquito human baiting rates to zero as reported for *Anopheles darling* after an intervention in Surinam [19]. The people interviewed have acquired this knowledge during more than a decade ago, which may have contributed to prevent the malaria transmission in the region. Official epidemiological data showed a reduction of malaria cases in Chiapas from 1995 to 2011, when people did not used to have access to impregnated bed nets as much as they do today (provided by some national programmes among them the Instituto Mexicano del Seguro Social [20]. Also, according to the 2006–2012 report from the Ministry of Health [21], 350 thousand ILLNs were distributed in the malarious areas in Mexico and although no cases have been reported in the study area, some villagers reported they were provided of such bed nets from some government programme. From the study of 1995 [10], 69.3% of the families reported to use some other methods as the production of smoke from burning flammable materials, to repel mosquitos. This kind of non-commercial method is more pronounced in rural areas than urban areas [22]. Nevertheless, the high percentage of villagers using any type of bed nets may represent an advantage for a high scale ITNs implementation programme. Furthermore, it is feasible that they would be keen to pay for the ITNs, if as they responded in the questionnaires, they have purchased any product to protect themselves from mosquito bites. In this study, no direct question was asked about whether the bed nets were traditional or impregnated. However, some of the interviewed people mentioned that they were provided of ITNs by some government programme (IMSS) but they still prefer not using insecticide-impregnated bed nets because of itchy skin reactions. This could contribute to a failure if ITN mass distribution is implemented for malaria elimination in the Mesoamerican region, although no other arguments against its use were mentioned, as the listed in other studies in Peru, where interviewed people reported their privacy being affected [23], as well as a perceived lack of effectiveness due to the size of the mesh [24]. A specific study to determine whether traditional bed nets are preferred as compared to ITNs would be needed in the Mesoamerican region to assess the impact of mass distribution if it is intended to be implemented.

During 1995, according to Rodriguez *et al*. [10] more than 86% of population interviewed reported that they had never contracted malaria, while the recently questionnaires applied showed an increment to 90.1% in the responses of households referring none of the family having malaria. This could be a good indicator of the success of the malaria control programme, but also could be a consequence of the improvement in the standard of living and socio economic level, and therefore more awareness on how to prevent malaria, including how to reduce human-vector contact. When trying to compare if the improvement of socio-economic factors could be directed linked to the referred reduction in people having malaria, a house condition and preventive practices indexes were built as reported in the methodology. These indexes were compared against reporting having malaria or not, as well as with the reported during the 1995 [10] study. No dependence of having or not malaria in the different levels of “house condition” of “preventive practices” was observed (*P* > 0.05).

In conclusion, although housing conditions, education level and malaria preventive practices have improved in these rural areas in 17 years [10], and the reports by interviewees of any member having malaria seem to be not associated with housing conditions (as measured only by walls, floors and roofs made of cement) or preventive practices (as measured only by bed nets possession and purchase of any product) an improvement of malaria control programmes needs to be implemented if the elimination of malaria is aimed in the region. Information generated by this study could help in the decision making about whether to use insecticide as indoor residual spraying or to implement massive distribution of long-lasting impregnated bed nets, considering the number of bedrooms and the structure of houses in the region, which may lead to a more efficient vector control for the coastal plain of Chiapas.

## Competing interests

The authors declare that they have no competing interests.

## Authors’ contributions

MMR interviewed households, collected the data in Excel, calculated percentages, filled the tables and wrote the draft of the manuscript. RPP was involved in the study design, interviewed households, started and revised manuscript drafts and conduct the discussion. JGO was involved in the study design, interviewed households and revised manuscript drafts. ADL and FS were involved in the study design and logistic and interviewed households. JLTE was involved in the statistical analysis and revised final manuscript. ADR was involved in the study design, direction of the people, interpretation of results, revised manuscript drafts and conduct the discussion. All authors read and approved the final manuscript.

## Supplementary Material

Additional file 1 KAP questionnaire applied to householders. Click here for file
