# Supplementary material for: Socioeconomic factors, attitudes and practices associated with malaria prevention in the coastal plain of Chiapas, Mexico
Source: Malar J. 2014 Apr 23;13:157. doi: 10.1186/1475-2875-13-157 (PMC4021278; doi:10.1186/1475-2875-13-157)
Supplement: Additional file 1 — KAP questionnaire applied to householders. [file 1475-2875-13-157-S1.pdf]

Annex 13.

I. Identification

1) Folio:

Municipality /_/_/ Village /_/_/ Family/_/_/_/

2) Date: /_/_/ Year /_/_/ Month /_/_/ Day

3) Village name /_/_/

____

4) Municipality /_/_/

____

5) Name of respondent /_/_/

____

II. Dwelling

6) Wall's surface (>50%) /_/
1. Block or brick
2. Wood
3. Plaster
4. Bamboo
5. Zinc
6. Cardboard or plastic
7. No walls
8. Other ___________________

7) Floor (>50%) /_/
1) Ground
2) Cement
3) Wood
4) Other___________________

8) Roof (>50%) /_/
1) Straw or palm
2) Cement
3) Wood
4) Zinc sheet
5) Tile
6) Other ___________________

9) Number of people living in the house /_/_/

10) Number ≤5 years old people living in the house /_/_/

11) Number of >5 years old people living in the house /_/_/

12) Number of bedrooms /_/

13) Average number of people sleeping per bedroom /_/

III. Symptoms, attitudes and preventive practices

14) Do you have bed nets? /_/
1. Yes
2. No (go to question 16)

15) Season in which you use bed nets /_/
1. Rainy season
2. Dry season
3. All year
4. Only when the abundance of mosquitoes increases
5. Ignore when
6. Never

16) Do you buy products for mosquito protection? /_/
1. Yes
2. No (go to question 18)

17) Number and name of productos you bought in the last year for mosquito protection

	1. Yes 2. No	(Total)
a) Bed nets	/_/	/_/_/
b) Insecticide in aerosol	/_/	/_/_/
c) Mosquito coils	/_/	/_/_/
d) Repellents	/_/	/_/_/
e) Other ____________	/_/	/_/_/

18) During the last week does any member of the family has shown any of the next symptoms?
1) Yes 2) No; Age: 1)<5 year, 2)>5 years, 3) Both groups (1 and 2)

	Yes/no	Age
a) Headache	/__/	/__/
b) Nauseas	/__/	/__/
c) Insensivity	/__/	/__/
d) Fuzzy vision	/__/	/__/
e) Vomiting	/__/	/__/
f) Diarrhea	/__/	/__/
g) Dizziness	/__/	/__/
h) Ringing in the ears	/__/	/__/
i) Back pain	/__/	/__/
j) Urethritis	/__/	/__/

4ª Norte Esq. con 19 Poniente
Tapachula, Chiapas
C. P 30700

tel: (52)-(962) 62 5 08 00
FAX: (52)-(962) 62 657-82
E-mail: americo@insp.com

k) Rhinorrhoea /__/ /__/
l) Abdominal pain /__/ /__/

19) Does any of your of your children under 5 year old got diarrhoea during the last 6 months? /__/
1.Yes
2. No

20) Does any member in the family has had malaria? /__/

1. Yes, one year ago
2. Yes, 2-5 years ago
3. Yes, 6-10 years ago
4. Yes, more tan 10 years ago
5. Nobody
6. Do not remember

21) How long since your house was last sprayed? /__/
1. Never
2. 1-2 months
3. 3-4 months
4. 5-6 months
5. 7-8 months
6. 9-12 months
7. More than one year ago

22) Do you agree if your house is sprayed periodically? /__/
1. Yes (go to question 24)
2. No

23) Why do you disagree?
____
____
____
(go to question 25)

24) How frequently would you like your house to be sprayed? /__/

1. Every 2 months
2. Every 3 months
3. Every 4 months
4. Every 6 months
5. Yearly

25) What is your schooling? /__/
1. Illiterate
2. Read and write
3. Elementary school incomplete
4. Elementary school
5. Middle school incomplete
6. Middle school
7. High school or higher

26) Occupation /__/
1. Farmer
2. Temporary worker
3. Trader
4. Fisherman
5. Student
6. Housewife
7. Other____________________

27) Do you use insecticide for agricultural or livestock purposes? /__/
1. Yes
2. No

28) Commercial name of the insecticide used
____
____
____
____
____

29) Frequency of insecticide usage during the year /__/
1. Every 2 months
2. Every 3 months
3. Every 4 months
4. Every 6 months
5. Yearly
6. Never apply

30) Do you apply the insecticide by yourself? /__/
1. Yes
2. No

4ª Norte Esq. con 19 Poniente
Tapachula, Chiapas
C. P 30700

tel: (52)-(962) 62 5 08 00
FAX: (52)-(962) 62 657-82
E-mail: americo@insp.com
